# Lebenslauforientierte Epidemiologie in der Migrationsforschung

**DOI:** 10.1007/s00103-023-03761-w

**Published:** 2023-09-15

**Authors:** Jacob Spallek, Hajo Zeeb, Oliver Razum

**Affiliations:** 1 Fachgebiet Gesundheitswissenschaften, Institut für Gesundheit, Brandenburgische TU Cottbus-Senftenberg, Senftenberg, Deutschland; 2 Lausitzer Zentrum für Digital Public Health, Brandenburgische TU Cottbus-Senftenberg, Senftenberg, Deutschland; 3 Leibniz-Institut für Präventionsforschung und Epidemiologie-BIPS, Bremen, Deutschland; 4 Health Sciences Bremen, Universität Bremen, Bremen, Deutschland; 5 AG3 Epidemiologie & International Public Health, Fak. für Gesundheitswissenschaften, Universität Bielefeld, Bielefeld, Deutschland; 6 Brandenburgische TU Cottbus-Senftenberg, Universitätsplatz 1, 01968 Senftenberg, Deutschland

**Keywords:** Lebenslaufepidemiologie, Migration und Gesundheit, Gesundheitliche Ungleichheit, Life course epidemiology, Migrant health, Health inequalities

## Abstract

Es gibt viele Gründe für Migration, von freier Entscheidung bis zu erzwungener Flucht. Entsprechend vielfältig sind auch die Vorgeschichten und Lebensumstände der migrierenden Menschen. Die damit einhergehenden unterschiedlichen Expositionen beeinflussen die Gesundheit der Migrant*innen und ihrer Kinder. Um ein solch komplexes Phänomen zu erfassen, ist ein Ansatz erforderlich, der die besonderen Umstände im Lebenslauf der Migrant*innen einbezieht.

Ein etablierter methodischer Ansatz, der dies leisten kann, ist die Lebenslaufepidemiologie. Bei der Anwendung dieses Konzepts auf migrierende Bevölkerungen werden Expositionen vor, während und nach der Migration untersucht. In der epidemiologischen Forschung zur Gesundheit von eingewanderten Menschen ist es wünschenswert, alle diese 3 Phasen zu berücksichtigen. Eine Herausforderung hierbei ist, dass verlässliche Daten über den gesamten Lebenslauf nicht immer verfügbar sind.

Eine valide, zeitnahe Erhebung und datenschutzgerechte Verknüpfung longitudinaler Daten aus verschiedenen Quellen können die lebenslaufbezogene Forschung zur Gesundheit von Migrant*innen in Deutschland verbessern. Perspektivisch sollten entsprechende Daten auch aus den Herkunftsländern von Migrant*innen einbezogen werden.

## Abstract (en)

From free choice to forced flight, there are many reasons for migration. Accordingly, the backgrounds and living conditions of migrating people are also diverse. The different associated exposures affect the health of migrants and their children. To capture such a complex phenomenon, an approach is required that takes specific living conditions during the life course of migrants into account.

An established methodological approach that can accomplish this is life course epidemiology. When applied to migrating populations, it examines exposures before, during, and after migration. In epidemiological research on the health of migrant populations, it is desirable to consider all three phases. This is countered by the fact that reliable data on the entire life course is not always available.

A valid, timely collection and data protection-compliant linkage of longitudinal data from different sources can improve life course-related research on the health of migrants in Germany. In the future, corresponding data should also be included from the countries of origin of migrants.

## Einleitung

Migration als weltweit und kontinuierlich auftretendes Phänomen betrifft in vielen Bereichen das Thema Gesundheit sowohl auf individueller als auch auf bevölkerungsbezogener Ebene [1, 2]. Aus epidemiologischer und Public-Health-Perspektive stehen dabei die Beschreibung der gesundheitlichen Situation, ihrer Veränderungen und möglicher Unterschiede zwischen Gruppen sowie die Erforschung möglicher Determinanten der Gesundheit im Vordergrund. Ziele sind, Risiken und Chancen, Gefahren und Potenziale für die Gesundheit bei Migrant*innen und ihren direkten Nachkommen sowie in der Bevölkerung insgesamt zu erforschen und Handlungsmöglichkeiten in Gesundheitsversorgung, -förderung und Prävention daraus abzuleiten.

Ein breites Spektrum von Perspektiven und Methoden ist zur Erforschung eines komplexen Phänomens wie der Migration und ihrer gesundheitlichen Folgen notwendig und sinnvoll. Ein etablierter und hilfreicher Ansatz in diesem Kontext ist die Lebenslaufepidemiologie (engl. Life Course Epidemiology), die aufgrund ihrer Betrachtung des gesamten Lebenslaufs eine strukturierte Analyse der jeweiligen Situation von Menschen mit ihren spezifischen Expositionen vor, während und nach der Migration beinhaltet [3, 4]. Dieser Beitrag beschreibt den Ansatz der Lebenslaufepidemiologie im Kontext von Migration und Gesundheit auf der Basis theoretischer und empirischer Arbeiten.

## Lebenslaufepidemiologie

Die Lebenslaufepidemiologie betrachtet Gesundheit als Ergebnis langfristiger biologischer, psychischer und sozialer Prozesse über den gesamten Lebenslauf von Menschen [5]. Etabliert hat sich dieser Ansatz in der Erforschung von Zusammenhängen zwischen frühkindlichen Risikofaktoren, wie z. B. einem geringen Geburtsgewicht, und einer frühzeitigen Morbidität und Mortalität im Erwachsenenalter [6]. Mittlerweile liegen zahlreiche empirische Belege für Auswirkungen von biologischen oder auch psychosozialen Risikofaktoren im Lebenslauf auf das Erkrankungsgeschehen im späteren Leben vor [7].

Die Interpretation und Erklärung dieser Zusammenhänge ist aufgrund der zeitlichen Abhängigkeiten und der Vielzahl beteiligter Faktoren komplex, vertiefende Literatur findet sich u. a. bei [5, 7, 8]. Zwei zentrale Konstrukte der Lebenslaufepidemiologie sind die „kritische Periode“ und die „Akkumulation von Expositionen“.

Unter „kritischen Perioden“ versteht man Phasen, in denen zentrale Weichenstellungen für die weitere gesundheitliche Entwicklung erfolgen, also physiologische und psychische Umbruchsphasen mit einer erhöhten Vulnerabilität gegenüber Störungen einer gesunden Entwicklung. Ein bekanntes und gut belegtes Beispiel für eine kritische Periode ist die Schwangerschaft, in der eine Vielzahl von Risiken für die langfristige gesunde Entwicklung des Babys durch äußere Faktoren besteht. So kann beispielsweise die Exposition gegenüber Schwermetallen nicht nur für die Mutter, sondern auch für die kindliche gesundheitliche Entwicklung langfristige Folgen haben [9].

Auch im weiteren Kindes- und Jugendalter gibt es Phasen, in denen wichtige Entwicklungsaufgaben stattfinden (z. B. Sprachentwicklung, Körperwachstum). Störungen in diesen Phasen wirken sich langfristig aus und können zu einem erhöhten Risiko für Krankheiten und Funktionseinschränkungen im fortgeschrittenen Alter führen. Ein Beispiel ist die Entwicklung des metabolischen Syndroms [7].

Unter der Risikoakkumulation versteht man, dass neben Ereignissen in den kritischen Perioden insbesondere auch kumulative und länger andauernde Prozesse die Gesundheit im Lebenslauf prägen [7, 10]. Beispiele sind eine dauerhaft nicht optimale Ernährung, dauerhafter Bewegungsmangel oder psychosoziale Belastungen wie chronischer Stress, die über die Jahre das Risiko z. B. für Herz-Kreislauf-Erkrankungen erhöhen können. Neben der Dauer kommt dabei auch der Intensität und der Kombination mehrerer Expositionen ein hoher Stellenwert zu. Für viele Risikofaktoren, wie z. B. geringe körperlich-sportliche Aktivität, Mangel- und Fehlernährung oder Rauchen, ist belegt, dass die Folgen für die Gesundheit umso stärker sind, je früher im Lebenslauf sie auftreten, je stärker sie ausgeprägt sind und je länger sie wirken [11].

## Lebenslaufepidemiologie in der Migrationsforschung

**Figure 1:**
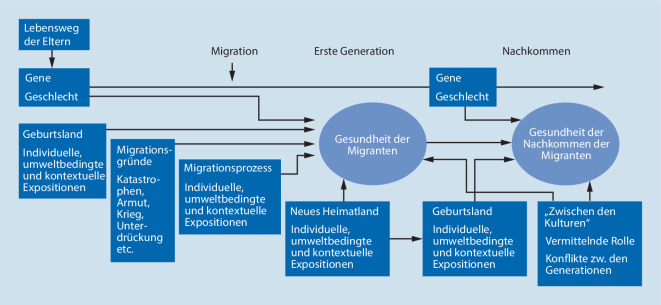

Kern des epidemiologischen Lebenslaufansatzes in der Forschung zu Migration und Gesundheit ist die Betrachtung des gesamten Lebenslaufes von Migrant*innen (und auch ihrer Kinder). Der Lebenslauf wird dabei in drei Abschnitte unterteilt: die Phase vor, während und nach dem Migrationsereignis (Abb. 1). In allen drei Phasen wirken Expositionen auf die Gesundheit der Migrant*innen, die sich von Menschen ohne Migrationserfahrung unterscheiden können.

Vorangestellt sei, dass eingewanderte Menschen eine sehr heterogene Gruppe und nicht generell kränker oder gesünder sind. Vielmehr unterscheiden sie sich von einer Bevölkerung ohne Migrationserfahrung hinsichtlich ihrer Gesundheit, den Risiken und gesundheitsförderlichen Ressourcen sowie spezifischen Bedarfen, je nach Herkunft, rechtlichem Status, sozioökonomischer Position, Bildung, Grund der Wanderung etc. [3, 4].

Nach dem Lebenslaufansatz resultiert die jeweilige individuelle gesundheitliche Situation zu verschiedenen Zeitpunkten im Leben aus einem spezifischen Auftreten und der Interaktion negativer und positiver gesundheitsrelevanter Faktoren über die Zeit. Vom jeweiligen Gesundheitsproblem, Zeit und Dauer des Auftretens der Exposition und gegebenenfalls kritischen Perioden im Lebenslauf hängt ab, welche Bedeutung sie für die Entstehung von Krankheiten im weiteren Lebenslauf haben. Manche gesundheitlichen Probleme entstehen aus einem Wechselspiel zwischen der Akkumulation von Risiken und der Prägung in für die Krankheitsentstehung besonders kritischen Perioden, z. B. während der Schwangerschaft oder Kindheit [3, 4].

Migrant*innen haben durch ihre Migrationserfahrung oft andere lebensgeschichtliche Expositionen als die nicht migrierte Bevölkerung. Diese Expositionen können sich, manchmal mit langer Latenzzeit, auf das Erkrankungsrisiko in späteren Lebensphasen – positiv oder negativ – auswirken. Daher ist eine Untersuchung des gesamten Lebenslaufs von Migrant*innen hilfreich, um ihre gesundheitliche Situation und ihre Risiken (wie beispielsweise für chronische Krankheiten) verstehen zu können. Eine Momentaufnahme der Gesundheit bzw. der Gesundheitsrisiken zu einem Zeitpunkt nach der Migration reicht hierzu meistens nicht aus [3–5].

In allen drei Phasen des Lebenslaufs – vor, während und nach dem Migrationsprozess – wirken Expositionen auf die Migrant*innen, die sowohl akut als auch im späteren Lebenslauf Einfluss auf die Gesundheit haben. Ein klassisches Beispiel für die zum Teil sehr lange Zeit zwischen Exposition und Auftreten der Krankheit ist die Beeinflussung des Risikos für das metabolische Syndrom im Erwachsenenalter durch perinatale und frühkindliche Programmierung des Stoffwechsels (sog. Barker-Hypothese; [6]). Ein weiteres Beispiel ist die Beeinflussung von Krebsrisiken durch die Exposition gegenüber Infektionen (z. B. Hepatitis-Viren und Leberkrebs, humane Papillomviren (HPV) und Zervixkarzinom, Helicobacter pylori und Magenkrebs). Entsprechende Expositionen sind in vielen Herkunftsländern häufiger als in Deutschland [12].

Die Phase vor der Migration kann daher besonders durch Expositionen gekennzeichnet sein, die sich erheblich von denen der nicht migrierten Bevölkerung unterscheiden [3]. Zu beachten sind dabei auch die Ereignisse, die den Ausschlag für die Migration gegeben haben, z. B. Natur- oder Umweltkatastrophen, Klimaveränderungen, Armut oder bewaffnete Konflikte. Aber auch Expositionen gegenüber spezifischen Krankheitserregern oder andere Lebensumstände, inkl. Ernährungssituation und Gesundheitsversorgung, können sich auf die spätere Gesundheit auswirken [3].

Eine besonders kritische Phase, insbesondere bei Geflüchteten, stellt der oft jahrelange Migrationsprozess dar, der von Stress, Ernährungs- und Hygieneproblemen bis hin zu Gewalt und sexueller Ausbeutung geprägt sein kann.

In der Phase nach der Migration werden dann Unterschiede im Gesundheitsverhalten bedeutsam, aber auch unterschiedliche Möglichkeiten gesellschaftlicher Teilhabe einschließlich der Zugänglichkeit zu (Gesundheits- und Sozial‑)Systemen, Diskriminierungserfahrungen sowie die besondere soziale Lage der Migrant*innen im Zielland (s. unten; [3, 4]).

Nicht nur die individuellen Gesundheitsrisiken sind einer zeitlichen Komponente unterworfen. Auch innerhalb von Migrant*innenpopulationen verändern sich Gesundheitsrisiken und Mortalität über die Zeit [12]. Im Vergleich zur nicht migrierten Bevölkerung höhere oder niedrigere Risiken können mit der Zeit konvergieren, da sich individuelle und kontextuelle Expositionen, soziale Teilhabe und Gesundheitsverhalten über den Lebenslauf verändern und innerhalb eines Kontextes oft angleichen.

Dies wurde beispielsweise für die Krebsrisiken von Frauen türkischer Herkunft in Hamburg gezeigt [13]. Frauen, die aus der Türkei zugewandert sind, haben signifikant niedrigere Risiken für Brustkrebs als die allgemeine weibliche Bevölkerung in Hamburg. Dieser Risikovorteil schwindet aber in den nachfolgenden Generationen. Als eine mögliche Ursache werden Veränderungen im reproduktiven Verhalten (wie Alter bei erster Geburt, Stillverhalten) vermutet [13, 14].

Ein weiteres Beispiel sind die durch Rauchen determinierten Risiken für Lungenkrebs. Während in der ersten Generation türkischer Migrant*innen das Rauchen unter Männern weitverbreitet war, haben Frauen deutlich seltener geraucht als die Männer, aber auch als deutsche Frauen, was sich in niedrigeren Risiken für Lungenkrebs widerspiegelte. Mit der Zeit hat hier eine Konvergenz stattgefunden: Die Häufigkeit des Rauchens bei türkischen Migrantinnen ist angestiegen [15] und damit auch ihr Lungenkrebsrisiko [13].

Auch Umweltbedingungen und das soziale Umfeld, als wichtige kontextuelle Determinanten der Gesundheit, können in den drei Phasen starken Veränderungen unterliegen. Dies trifft ebenso für Verfügbarkeit, Qualität und Zugang der Krankheitsversorgung zu. Ein weiterer wichtiger Aspekt der Gesundheit ist die individuelle sozioökonomische Position, die bei Menschen mit und ohne Migrationsgeschichte eine zentrale Determinante der Gesundheit darstellt, über den gesamten Lebenslauf wirkt und sich über die Zeit stark verändern kann. Das Zusammenspiel von Migration und sozialer Lage als mögliche Determinanten gesundheitlicher Ungleichheit wird in einem eigenen Abschnitt weiter unten ausführlich diskutiert.

Durch im Vergleich zur Bevölkerung ohne Migrationserfahrung unterschiedliche Expositionen in den Phasen vor, während und nach der Migration entsteht die besondere gesundheitliche Situation von Migrant*innen sowohl im Herkunfts- als auch im Zielland. Eine Berücksichtigung aller drei Phasen ist daher für die Einordnung und Bewertung der Gesundheit von Migrant*innen bedeutsam, auch wenn oftmals verlässliche Daten über den gesamten Lebenslauf nicht verfügbar sind.

## Die Nachkommen der eingewanderten Menschen

Das Lebenslaufmodell von Migration und Gesundheit (Abb. 1) zeigt, dass sich die gesundheitlichen Auswirkungen einer Migration auch auf die Kinder von Migrant*innen erstrecken können. Auch diese sogenannte zweite Generation befindet sich oftmals noch in einer durch die Migration ihrer Eltern beeinflussten gesundheitlichen Situation [4, 16]. Das kann die materielle und soziale Lebenslage betreffen, in der Kinder aufwachsen, aber auch die Prägung von Verhaltensweisen, wie z. B. Essgewohnheiten oder den Umgang mit Gesundheit und dem Gesundheitssystem [4, 16].

Biologische und soziale gesundheitsrelevante Faktoren können sich von der ersten Generation auf die zweite Generation übertragen. So ist beispielsweise ein dunklerer Hauttyp genetisch determiniert. Wird er von den Eltern an die Kinder weitergegeben, so beeinflusst dies auch Gesundheitspotenziale und -risiken, wie z. B. ein geringeres Risiko, an Hautkrebs zu erkranken. Gleichzeitig kann dadurch die gesundheitliche Lage beeinflusst werden, etwa wenn aus einer dunkleren Hautfarbe oder anderen Merkmalen eine individuelle oder gesellschaftliche Diskriminierung resultiert [17, 18]. Die Arbeit von Akbulut und Razum in diesem Themenheft führt dieses Thema weiter aus.

Eine Weitergabe von gesundheitlichen Vorteilen und Risiken geschieht aber nicht nur genetisch, sondern auch sozial: Kinder übernehmen oftmals Verhaltensweisen von ihren Eltern, z. B. im Bereich der Ernährung, der körperlichen Aktivität oder des Konsumverhaltens. Spezifische Verhaltensweisen können ein wichtiges Instrument zur Wahrung der eigenen Identität sein und sich über Generationen von denen in der deutschen Bevölkerung ohne Migrationserfahrung unterscheiden – bis hin zu einer bewussten Abgrenzung – oder sie werden schnell abgelegt, z. B. um in der Gesellschaft nicht aufzufallen [19]. Dabei zeigt sich ebenfalls der prozesshafte und zeitliche Charakter von Migration: Migration und gesellschaftliche Integration sind geprägt durch kontinuierliche Aushandlungsprozesse und sich verändernde gesellschaftliche Rahmenbedingungen.

Aufgrund dieser Aushandlungs- und Teilhabeprozesse und weiteren sozialen und physischen Expositionen im Zielland der Migration kann sich die zweite Generation der Migrant*innen gesundheitlich von Menschen ohne Migrationserfahrung in der Familienbiografie, aber auch von ihren Eltern unterscheiden. Ein Beispiel ist die weiter oben beschriebene aus gesundheitlicher Sicht negative Konvergenz von Krebsrisiken [4, 16].

Die direkten Nachkommen von Menschen mit Migrationserfahrung können zudem nicht nur beeinflusst sein durch die Migration ihrer Eltern, sondern sie prägen auch selbst die Gesundheit in ihrer Umgebung und beeinflussen beispielsweise die Gesundheit ihrer Eltern und Kinder [4]. Dabei können sie als Mediator*innen und Vermittler*innen auftreten und gesundheitliche Belange ihrer Eltern unterstützen und vertreten [4, 16].

Migrant*innen und ihre Nachfahren sind Teil der Gesellschaft und nehmen aktiv Einfluss. Viele Menschen mit eigener oder familiärer Migrationserfahrung arbeiten z. B. im Gesundheits- und Sozialwesen und bringen dadurch ihre Kompetenzen und Perspektiven in die Gestaltung (z. B. der Gesundheitsversorgung) ein. Auch hier zeigt sich, dass „Migration“ von der Gesundheitsforschung in einem Einwanderungsland bei Weitem nicht nur als ein spezifischer Risikofaktor, sondern als ein multidimensionales Phänomen mit weitreichenden gesundheitlichen Chancen, Risiken und Entwicklungsmöglichkeiten betrachtet werden muss.

Aus der Sicht von Public Health stellt die zweite Generation also eine Bevölkerungsgruppe mit möglicherweise anderen gesundheitlichen Risiken, aber auch einer Vielzahl von Potenzialen dar, die sich von der selbst zugewanderten Generation unterscheidet. Auch an dieser Stelle ist das Wechselspiel von Auswirkungen des Sozialstatus auf die Gesundheit – die migrierte und nicht migrierte Menschen betreffen – mit den Auswirkungen der Migration auf die Gesundheit zu beachten (s. unten).

Über wie viele Generationen Auswirkungen der Migration anhalten, ist noch nicht ausreichend untersucht. Das liegt nicht nur an den erforderlichen langen Beobachtungszeiträumen. Es stellt sich die Frage, ob eine fortlaufende Zuschreibung der familiären Migrationsgeschichte sinnvoll oder nicht sogar hinderlich ist, um die gesundheitliche Situation der dritten Generation adäquat zu erfassen [20]. In Großbritannien beispielsweise steht der Begriff der „ethnischen Minoritäten“ im Vordergrund, deren gesundheitliche Situation sich aufgrund ähnlicher Determinanten wie bei Migrant*innen von der der Bevölkerung ohne Migrationserfahrung unterscheiden kann, ohne dass hier die Migration an sich das definierende und in Statistiken erfasste Merkmal ist [21].

## Lebenslaufbezogene Analyse der gesundheitlichen Ungleichheit

In der Lebenslaufepidemiologie zeigt sich ein starker Zusammenhang zwischen sozialer Position und Gesundheit [22]. Dieser soziale Gradient begründet sich dadurch, dass zahlreiche gesundheitliche Belastungen, oftmals schon in frühen Lebensphasen, sozial ungleich verteilt sind. So sind Kinder aus ärmeren und geringer gebildeten Familien z. B. häufiger von kindlichem Übergewicht oder Entwicklungsstörungen im Kleinkindalter betroffen [23]. Diese als „gesundheitliche Ungleichheit“ bezeichnete sozial ungleiche Verteilung von Risikofaktoren schreibt sich meistens bis ins hohe Alter fort. Sozial benachteiligte Personen haben über den gesamten Lebenslauf erhöhte gesundheitliche Risiken und damit verbundene gesundheitliche Beeinträchtigungen [22].

Manchmal wird argumentiert, gesundheitliche Nachteile von Migrant*innen nach der Migration seien weitgehend eine Folge des niedrigen sozialen Status [4, 22]. Tatsächlich haben Migrant*innen überproportional häufig einen niedrigen Sozialstatus und im Durchschnitt häufiger eine schlechtere Schulbildung und berufliche Qualifikation als die nicht migrierte Bevölkerung im Zielland [16, 24]. Eine höhere Arbeitslosen- und Armutsquote sowie im Durchschnitt geringere Einkommen sind die Folgen.

Hinzu kommen manchmal, insbesondere bei Männern mit Migrationserfahrung, statusspezifische gesundheitsschädigende Verhaltensweisen, wie beispielsweise ein höherer Anteil an Rauchern [24]. Diese Belastungen und Risiken können mit schlechterer Gesundheit, besonders in zunehmendem Alter, assoziiert sein [25]. Zu beachten ist dabei aber, dass nicht migrierte Männer mit gleichem Sozialstatus ähnliche Belastungen und Verhaltensweisen aufweisen und damit auch vergleichbaren gesundheitlichen Risiken unterliegen [25]. Es ist daher wichtig, zwischen statusspezifischen und migrationsspezifischen Ursachen zu trennen. Beide jedoch spielen für die Gesundheit von Migrant*innen eine wichtige Rolle.

Aus sozialepidemiologischer Sicht ist eine eigene Migrationserfahrung, bzw. die der Eltern, ein wichtiger Faktor für die Beschreibung der gesundheitlichen Ungleichheit einer Bevölkerung [22]. Die Verbindung von Migration und Gesundheit ist dabei multidimensional und die Migrationserfahrung (manchmal Migrationshintergrund genannt) fungiert als eine Art Surrogat für eine Vielzahl von damit verbundenen Mechanismen und Prozessen [4, 16]. Ohne Berücksichtigung des sozialen Status ist die Analyse der gesundheitlichen Situation von Migrant*innen meist nur von begrenztem Aussagewert [4].

Weitere Bedeutung bekommen diese Prozesse durch ihre Interaktion mit der sozialen Mobilität. Kinder aus sozial benachteiligten Familien haben nicht nur ein höheres Risiko für gesundheitliche Belastungen, sondern sind zugleich auch im Hinblick auf ihre Bildungschancen benachteiligt [22]. Im deutschen Bildungssystem hängt der Bildungserfolg weiterhin in starkem Maße von der sozioökonomischen Position der Herkunftsfamilie ab [26]. Erreichen Jugendliche dadurch ihrerseits nur niedrigere Bildungsabschlüsse, so haben sie mit einer großen Wahrscheinlichkeit auch in ihrem weiteren Leben eher niedrigere berufliche Positionen mit niedrigeren Einkommen. Über die Verbindung hiervon mit diversen negativen gesundheitlichen Belastungen schreiben sich somit soziale und gesundheitliche Benachteiligungen fort [27].

Diese Fortschreibung gesundheitlicher Ungleichheit ist einerseits unabhängig von einer Migrationserfahrung, andererseits können Familien mit Migrationserfahrung, z. B. aufgrund besonderer Barrieren (Sprache, Kenntnisse des Bildungssystems etc.), besonderen Risiken unterliegen, deren Beachtung unter dem Aspekt der Chancengleichheit eine komplexe, aber auch wichtige Herausforderung darstellt [28].

Auch bei der sogenannten Vererbung gesundheitlicher Ungleichheit gibt es neben den genannten sozialen auch migrationsspezifische Prozesse, die Inhalt aktueller Forschung sind. So erleben Frauen mit türkischem Migrationshintergrund in Deutschland höheren subjektiven Stress und häufiger depressive Symptome während der Schwangerschaft, einhergehend mit höheren Entzündungswerten und einer schwächeren Cortisol-Aufwachreaktion im Vergleich zu Frauen ohne Migrationshintergrund [29–31]. Höhere Entzündungswerte und eine veränderte Stressreaktion der Mutter während der Schwangerschaft können sich negativ auf die Stressbiologie des Kindes auswirken. Diese Ergebnisse unterstützen somit die Hypothese, dass migrationsbezogene Erfahrungen wie Stress, soziale Deprivation oder Diskriminierung [17] sich über die Stressbiologie in der Schwangerschaft übertragen und damit auf die folgende Generation auswirken können. Inwieweit hiermit langfristige, lebenslaufbezogene gesundheitliche Konsequenzen verbunden sind, wird Gegenstand weiterer Forschung sein müssen.

## Diskussion und Fazit

Die Lebenslaufepidemiologie in der Migrationsforschung betrachtet die Gesundheit von Menschen mit Migrationserfahrung und ihren Nachkommen aus einer gesamtheitlichen Perspektive über den Lebenslauf. Der komplexe und multidimensionale Zusammenhang zwischen Migration und Gesundheit kann am besten abgebildet werden, wenn die vielfältigen zugrunde liegenden Prozesse adäquat in epidemiologische Studien und daraus abgeleitete Erklärungsmodelle integriert werden [3, 4, 16]. Zudem ist eine zeitliche Komponente, sowohl individuell in der Lebenslaufperspektive als auch für die Beschreibung von Veränderungen über Gruppen und Generationen, notwendig.

Die eingewanderte Bevölkerung ist vielfältig und bildet unzählige individuelle Lebenswege aus zahlreichen Herkunftsländern ab. Sie verändert sich ständig – über die Zeit und durch neue Migrationsbewegungen und Niederlassungsprozesse. Analysen und Erklärungsmodelle sind allerdings oft nur eine Darstellung zu einem bestimmten Zeitpunkt. Sie müssen daher kontinuierlich an die aktuelle Situation angepasst und auf der Basis neuer empirischer Ergebnisse und theoretischer Überlegungen weiterentwickelt werden.

Vorliegende empirische Ergebnisse zur gesundheitlichen Ungleichheit im Lebenslauf – auch bei eingewanderten Menschen – resultieren überwiegend aus großen Kohortenstudien aus dem angloamerikanischen und skandinavischen Raum. Auch die hierzulande zunehmend bessere Datenlage sollte vermehrt für längsschnittliche, lebenslaufbezogene Analysen genutzt werden, insbesondere für eingewanderte Menschen und ihre Kinder. Solche Analysen haben nicht nur einen Wert für die Grundlagenforschung, sondern helfen bei der Identifikation spezifischer Risiko- oder Ressourcenkonstellationen in einzelnen Lebensphasen, auf die eine zielgerichtete Prävention gelenkt werden könnte.

Eine idealtypische epidemiologische Studie zum Thema Migration und Gesundheit würde längsschnittlich nicht nur Migrant*innen und ihre Nachfahren in ihrem neuen Heimatland, sondern auch Menschen in den Herkunftsländern und Migrant*innen in anderen Zielländern einschließen [3, 32]. Somit würden längsschnittliche Vergleiche gesundheitlicher Verläufe von Menschen mit und ohne Migrationserfahrung in verschiedenen Zielländern mit Menschen im Herkunftsland möglich, wodurch eine genauere Identifizierung und Differenzierung migrationsspezifischer und sozialer Gesundheitsdeterminanten in den jeweiligen Ländern ermöglicht würde. Über die Generationen werden zudem die Gesellschaften der Einwanderungsländer diverser, auch die Potenziale dieser Entwicklung und ihr zumeist positiver Einfluss auf Public Health sollten verstärktes Ziel zukünftiger Forschung werden.

Migrant*innen und ihre direkten Nachkommen, eine relevante und große Gruppe der Bevölkerung, sollten routinemäßig in epidemiologische Analysen einbezogen werden. Dabei gilt es, ihre spezifischen Lebensläufe zu berücksichtigen. Oftmals wird dies durch eine unzureichende Datenlage erschwert, da in vielen Studien eingewanderte Menschen immer noch nicht entsprechend ihrem Anteil in der Bevölkerung einbezogen werden. Andererseits könnten die vorhandenen Daten, die z. B. durch die NAKO-Gesundheitsstudie (https://nako.de/) [33], das Sozio-oekonomische Panel (SOEP) oder den Mikrozensus bereitgestellt werden, noch häufiger genutzt werden.

Deutschland spielt eine wichtige Rolle bei der Aufnahme von Geflüchteten aus Krisenregionen. Defizite bei der zeitnahen Bereitstellung von Daten zur gesundheitlichen Situation dieser Menschen werden bereits lange bemängelt [34]. Viele Geflüchtete werden langfristig in Deutschland bleiben und haben spezifische Gesundheitsprobleme und Versorgungsbedarfe (s. andere Beiträge in diesem Themenheft). Um diese Defizite in der Datenlage abzubauen, müssen Zugänge für eine zeitnahe valide Gewinnung von Daten zur gesundheitlichen Situation entwickelt werden, insbesondere für Personen, die gerade erst nach Deutschland gekommen sind. Die dabei gewählten Definitionen der Zielgruppen dürfen nicht diskriminierend sein [35]. Sprachliche Barrieren müssen Berücksichtigung finden und verwendete Instrumente müssen validiert sein. In dieser Hinsicht gehen Initiativen wie RESPOND (https://respond-study.org) und IMIRA^1^ wichtige Schritte in die richtige Richtung. Um die Lebenslaufforschung für eingewanderte Menschen in Deutschland voranzutreiben, wäre eine datenschutzgerechte und qualitätsgesicherte Zusammenführung longitudinaler Daten, auch unter Einbezug der Herkunftsländer, wünschenswert.

## Footnotes

1 www.rki.de/imira (letzter Zugriff 30.05.2023).
